# Recovery of Citric Acid from Fermentation Broth Using Supported Ionic Liquids

**DOI:** 10.1021/acsomega.6c04343

**Published:** 2026-10-20

**Authors:** Rachel de Moraes Ferreira, Márcia C. Neves, Maria Alice Zarur Coelho, Bernardo Dias Ribeiro, Mara G. Freire

**Affiliations:** † Department of Biochemical Engineering, School of Chemistry, Federal University of Rio de Janeiro, Rio de Janeiro 21941-909, Brazil; ‡ CICECO—Aveiro Institute of Materials, Chemistry Department, University of Aveiro, Aveiro 3810-193, Portugal

## Abstract

Citric acid is widely used in the pharmaceutical, food, and cosmetic industries. However, the recovery of bioproduced citric acid from fermentation broths typically involves purification steps that generate substantial waste. This work proposes an alternative recovery strategy based on the selective adsorption of citric acid using supported ionic liquids (SILs). Two SILs were prepared by immobilizing the IL ligands diethylpropylethanolammonium chloride ([Si]­[N_3222OH_]­Cl) and 1-propyl-3-methylimidazolium chloride ([Si]­[C_3_C_1_Im]­Cl) onto a solid silica support. The materials were characterized by ^13^C NMR spectroscopy, elemental analysis, and determination of the specific surface area (*S*_BET_). Batch adsorption experiments were conducted to evaluate the effects of contact time, initial citric acid concentration, temperature, and pH. Adsorption kinetics were analyzed using pseudo-first-order, pseudo-second-order, intraparticle diffusion, and Boyd models, while equilibrium data were interpreted using the Langmuir, Freundlich, and Dubinin–Radushkevich isotherm models. The pseudo-second-order model best described the adsorption kinetics, whereas the Freundlich model provided the best fit for the equilibrium data. Maximum adsorption capacities of 577 and 793 mg·g^–1^ were obtained for [Si]­[N_3222OH_]Cl and [Si]­[C_3_C_1_Im]­Cl, respectively. Reusability of the SILs was also assessed through successive adsorption–desorption cycles. The SILs enabled a citric acid desorption range of 43.8% to 100%. Finally, the materials were applied to the recovery of citric acid directly from a fermentation broth to evaluate their potential for industrial application. Overall, these results highlight the potential of SILs as promising adsorbent materials for the efficient recovery of citric acid from fermentation broths, paving the way for their widespread use at the industrial scale.

## Graphical abstract

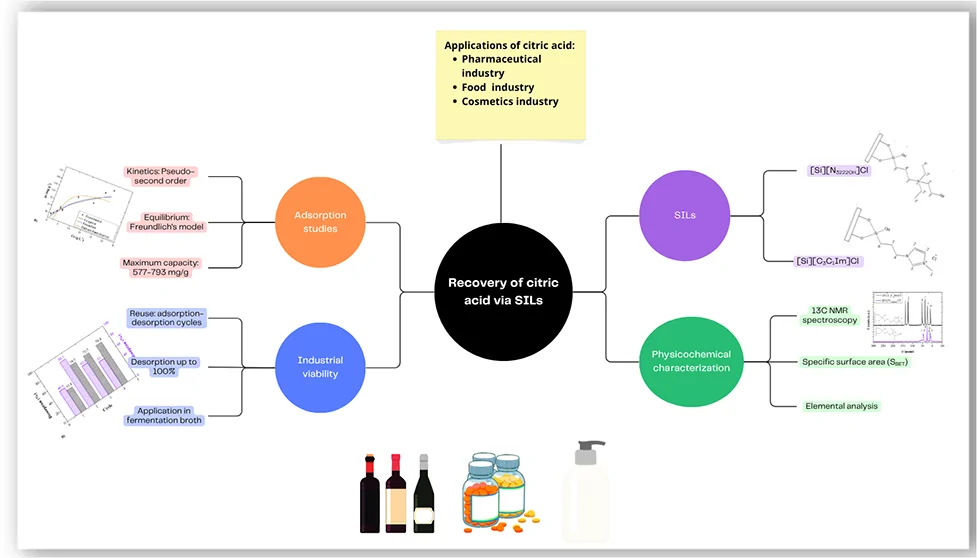

## 1. Introduction

Citric acid is a naturally occurring organic acid widely distributed in living organisms. It is particularly abundant in citrus fruits, where it contributes to their characteristic acidity. In addition to these high concentrations in fruits, citric acid is present in smaller amounts in many plants, animals, and microorganisms because it plays a central role as an intermediate metabolite in the Citric Acid Cycle (Krebs cycle), a fundamental pathway of cellular respiration.^1–3^ Owing to its natural occurrence, high solubility in water, and recognized safety, citric acid is extensively employed in the food, pharmaceutical, and cosmetic industries, where it functions as a pH regulator, preservative, emulsifying and dispersing agent, and chelating agent, among other applications.^4^ Citric acid has low toxicity and is currently considered safe for consumption by the Food and Drug Administration (FDA), in addition to being biodegradable.^5^

At present, citric acid is predominantly produced at an industrial scale through microbial fermentation processes, which represent the main route for its commercial manufacture. However, fermentation broths are complex mixtures that contain not only citric acid but also several other components, including microbial biomass, residual nutrients, mineral salts, proteins, and amino acids. The presence of these compounds increases the complexity of downstream processing, making the separation and purification of citric acid a challenging and critical step in the overall production process.^2,3,6^

The conventional method for purifying citric acid from fermentation broths relies on its precipitation as tricalcium citrate through the addition of calcium carbonate. The precipitate is then separated by filtration, followed by acidification with sulfuric acid to regenerate citric acid and the subsequent evaporation of the solution to obtain the final product. However, this process involves multiple steps and the consumption of large amounts of chemicals, which increases operational costs and generates significant quantities of waste, particularly calcium sulfate as a byproduct.^2,3,6^ To overcome these limitations, several alternative separation and purification strategies have been investigated, including electrodialysis, ultrafiltration and nanofiltration, liquid membrane separation, and adsorption-based processes.^3,7^

Adsorption has emerged as a promising alternative for the purification of citric acid. In addition to offering a certain degree of selectivity, adsorption-based processes may reduce waste generation and simplify downstream processing. Moreover, depending on the adsorbent employed, this approach can represent an economically advantageous separation strategy.^8^ In this context, the present work proposes the use of silica modified with ionic liquids, namely supported ionic liquids (SILs), as effective adsorbent materials for the purification of citric acid from the fermentation broth.

SILs have demonstrated promising performance as adsorbent materials for a wide range of compounds, including metal ions, nucleic acids, acetylsalicylic acid, sodium diclofenac, anticancer drugs, and other organic molecules.^9–16^ Beyond the specific application to citric acid, the novelty of the present work lies in the systematic evaluation of both adsorption and desorption processes aimed at enabling the efficient recovery and purification of citric acid. In particular, this study assesses the adsorption performance of citric acid on SILs through consecutive adsorption–desorption cycles, allowing the evaluation of adsorbent recyclability and material stability. Furthermore, additional adsorption experiments were conducted using fermented broth to investigate the performance of the materials under more realistic and complex conditions relevant to citric acid production processes.

## 2. Methodology

### 2.1 Preparation of SILs

The SILs investigated correspond to silica modified with diethylpropylethanolammonium chloride ([Si]­[N_3222OH_]­Cl) and silica modified with and 1-propyl-3-methylimidazolium chloride ([Si]­[C_3_C_1_Im]­Cl), which were synthesized following the method described by Bernardo et al.,^10^ yet with some modifications. In the present study, a fixed amount of 0.035 mol of precursor (*N*,*N*-Diethylethanolamine or *N-*methylimidazole) was used for every 5 g of the starting material. Briefly, 5 g of silica was previously activated with hydrochloric acid over 24 h. After that, it was washed with distilled water until achieving a neutral pH. After this step, 50 mL of toluene and 5 mL of (3-chloropropyl)­triethoxysilane were added. This suspension was left in reflux at 105 °C under agitation for 24 h. The suspension was then filtered using a vacuum glass filter as soon as the temperature dropped to room temperature, followed by a washing step with toluene, ethanol: water (1:1, v/v), distilled water, and methanol. The resultant materials were dried at 60 °C for at least 24 h. The obtained dried 3-chloropropyl silica precursor ([Si]­[C_3_]­Cl) was added to a 4.6 mL of *N*,*N*-Diethylethanolamine in 50 mL of toluene mixture. This suspension was refluxed at 105 °C under magnetic agitation for 24 h. Next the suspension was filtered and washed with toluene, methanol, distilled water, and methanol, followed by a drying step at 60 °C for at least 24 h. This procedure yielded the SIL [Si]­[N_3222OH_]­Cl. The same method was used to synthesize the [Si]­[C_3_C_1_Im]­Cl, using 2.8 mL of *N*-methylimidazole.

### 2.2 Characterization of SILs

The SIL characterization comprised solid-state ^13^C NMR (Carbon-13 Nuclear Magnetic Resonance), were performed at room temperature using a Bruker Avance III-400 MHz spectrometer (DSX model) with 4 mm BL cross-polarization magic angle spinning (CPMAS) VTN probes at 100.6 MHz. 100 mg was placed in a ZrO_2_ rotator of 4 mm diameter. In order to increase the signal-to-noise ratio of the solid-state spectra, the CPMAS NMR ^13^C settings used were the following: V1 13C 1/4 55 kHz; recycle delay, 4 s; contact time,1–2 ms; NS 1/4 1 k; VR 1/4 12 kHz.

The Brunauer–Emmett–Teller specific surface area (*S*_BET_) of SILs was evaluated by gas adsorption using a Gemini V-2380 surface area analyzer (Micromeritics, Norcross, GA, USA) and determined by the Brunauer–Emmett–Teller (BET) equation with multipoint adsorption isotherms of N_2_ at −196 °C. Prior to the measurements, the samples were degassed overnight at 100 °C.

The content (weight percentage) of carbon, hydrogen, and nitrogen was determined by elemental analysis using a TruSpec 630–200–200 CHNS analyzer. 2 mg of sample was introduced into the apparatus at a combustion furnace temperature of 1075 °C and an afterburner temperature of 850 °C. Infrared absorption was used to determine the carbon and hydrogen contents, whereas thermal conductivity was used for nitrogen quantification.

#### 2.2.1 Bonding Density

The intermediate silica bonding density was determined from the silica gel *S*_BET_ value according to the work of Yang et al.^17^ applying eq 1

1 $$bonding\,\;density\;\left(\mu \frac{mol}{m^{2}}\right)=\frac{\%N\times 10^{6}}{{{n\times M}_{N}\times S}_{BETsilica}}$$

where %*N* is the percentage of SIL nitrogen obtained in the elemental analysis, *M*_N_ is the molar mass of nitrogen, *n* is the number of nitrogen present in the SIL molecule, and *S*_BETsilica_ is the surface area of silica. In the case of [Si]­[N_3222OH_]­Cl, *n* is equal to 1, and for [Si]­[C_3_C_1_Im]­Cl, *n* is equal to 2 due to the number of nitrogen atoms present in each IL molecule.

### 2.3 Adsorption Kinetics and Isotherms

Commercial citric acid aqueous solutions were prepared to carry out initial adsorption kinetics and isotherms. For kinetic tests, 100 mL of a citric acid solution (10 g·L^–1^) was placed in contact with 500 mg of each SIL ([Si]­[N_3222OH_]Cl or [Si]­[C_3_C_1_Im]­Cl), and kept under stirring at 120 rpm, at room temperature (25 ± 1 °C). Aliquots were taken at previously established intervals (0 to 180 min). Samples were centrifuged at 9000 rpm for 5 min and filtered for quantification of the citric acid concentration by high-performance liquid chromatography (HPLC). Solutions without SILs were used as a negative control (blank) to confirm and determine the initial concentration. All experiments were performed in triplicate.

For the adsorption isotherms, 5 mL of solutions with citric acid concentrations ranging from 0.1 to 30 g·L^–1^ were used, added to 50 mg of each SIL ([Si]­[N_3222OH_]Cl or [Si]­[C_3_C_1_Im]­Cl), under stirring at 120 rpm for 30 min. After this period, the samples were centrifuged (9000 rpm, 5 min), filtered, and analyzed by HPLC. The experimental data were fitted to the Langmuir, Freundlich, and Dubinin–Radushkevich models. All tests were performed in triplicate.

### 2.4 Effect of Temperature and pH on the Citric Acid Adsorption

To study the influence of temperature, 50 mg of each SIL was mixed with 1 mL of citric acid solution (10 g·L^–1^), under stirring at 120 rpm for 30 min at different temperatures (25, 30, 35, and 40 °C). The samples were centrifuged (9000 rpm, 5 min), and the supernatant was filtered and analyzed by HPLC. The same procedure was carried out to evaluate the effect of pH at 2, 3, 4, and 5, adjusted with 0.1 M HCl and 0.1 M NaOH, while maintaining room temperature. All experiments were conducted in triplicate.

### 2.5 Adsorption Efficiency in Consecutive Adsorption and Desorption Cycles

Silica regeneration was evaluated through desorption experiments. Initially, 100 mg of SILs were saturated with 10 mL of a citric acid solution (10 g·L^–1^) under stirring at 120 rpm for 30 min at room temperature. After centrifugation (9000 rpm, 5 min), the supernatant was separated for analysis.

Then, 5 mL of a 0.1 mol L^–1^ NaOH solution was added, followed by stirring at 120 rpm for 5 min. The system was centrifuged again (9000 rpm, 5 min), and the supernatant was separated. Subsequently, 5 mL of a 0.1 mol L^–1^ HCl solution was added to the supernatant to adjust the pH. Citric acid was quantified by HPLC.

Next, 5 mL of HCl solution was added to the SILs and stirred for 10 min, followed by centrifugation and removal of the supernatant. The SILs were washed three times with deionized water and dried at 60 °C for 24 h. The cycle was repeated several times to evaluate the regeneration capacity of the SILs.

### 2.6 Recovery of Citric Acid from the Fermentation Broth

To evaluate adsorption in real media, 50 mg of each SIL was added to 5 mL of fermented broth containing citric acid and kept under stirring at 120 rpm for 30 min at room temperature. After this period, the samples were centrifuged at 9000 rpm for 5 min, and the supernatant was filtered and quantified by HPLC. All experiments were performed in triplicate. For desorption assays, the previously described procedure was applied.

### 2.7 Microbial Cultures

The *Yarrowia lipolytica* IMUFRJ 50682 strain was initially cultivated in preinoculum medium containing yeast extract (10 g·L^–1^), peptone (20 g·L^–1^), and glucose (20 g·L^–1^), under agitation at 160 rpm for 48 h at 28 °C. An aliquot of the culture was centrifuged and the biomass resuspended in 50 mL of mineral minimum medium composed of (g·L^–1^): (NH_4_)_2_SO_4_ (3.0000), KH_2_PO_4_ (1.0), K_2_HPO_4_ (0.1600), MgSO_4_·7H_2_O (0.7000), NaCl (0.5), Ca­(NO_3_)_2_·4H_2_O (0.4000), thiamine hydrochloride (0.0003), glycerol (30.0) and yeast extract (0.1705). The culture was kept under agitation at 120 rpm for 96 h at 28 °C. After this period, the fermented broth was centrifuged at 9000 rpm for 10 min to separate the cells, with the supernatant being used in the tests described in Section 2.6.

### 2.8 Citric Acid Quantification

The citric acid concentration was determined by high-performance liquid chromatography (HPLC), using a double pump system and UV detector with zinc lamp, operating at 210 nm. The column used was C18 (5 μm, 250 mm × 4.6 mm, pore 1000 Å), in isocratic mode with mobile phase composed of 0.1% (v/v) acetonitrile and 0.125% (v/v) phosphoric acid, at a flow rate of 0.6 mL.min^–1^. The injection volume was 20 μL, with wavelength set to 210 nm. The oven temperature was maintained at 26 °C.^18,19^ Under these conditions, citric acid showed a retention time of approximately 7 min, and the total run time for each analysis was 20 min.

## 3. Results and Discussion

### 3.1 Characterization of SILs

The [Si]­[C_3_C_1_Im]Cl was synthesized according to a previously published protocol.^10^ Thus, ^13^C NMR, elemental analysis, and the *S*_BET_ surface area assays were performed to verify whether the silica functionalization was successful. In the ^13^C NMR spectra provided in Figure 1, it is possible to observe the signals for [Si]­[C_3_C_1_Im]Cl at 47, 27, and 10 ppm, corresponding to the three carbon atoms of the propyl alkyl chain (C4, C5, and C6), the peaks ranging from 120 to 140 ppm, corresponding to the aromatic carbons of imidazolium (C3 and C2), and the signal at 37 ppm, corresponding to the carbon of the methyl chain (C1). These peaks confirm the functionalization of silica with the imidazolium-based ligand, being in agreement with the literature.^10^ The [Si]­[N_3222OH_]Cl was here synthesized and characterized for the first time. The respective ^13^C NMR spectrum shows peaks at 27 and 10 ppm, referring to two carbon atoms of the propyl alkyl chain (C4′ and C5′), and the peak at 11 ppm corresponding to the third carbon of the methyl chains (C3′). The peaks referring to the carbons of the alkyl chain (C1′ and C2′) are convoluted at 46 ppm. According to the Spectral Database for Organic Compounds (SDBS), the ^13^C NMR SDBS No. 1887 Spectral code: CDS-00–816 for the molecule 2-(diethylamino)­ethanol presents a signal at 11.88 ppm referring to the methyls (C3′), at 47.02 and 54.82 ppm referring to the carbons of the alkyl chain (C2′), and at 58.81 ppm for the carbon linked to the hydroxyl (C1′).^20^ These results indicate that the successful functionalization of [Si]­[N_3222OH_]Cl was carried out.

**Figure 1:**
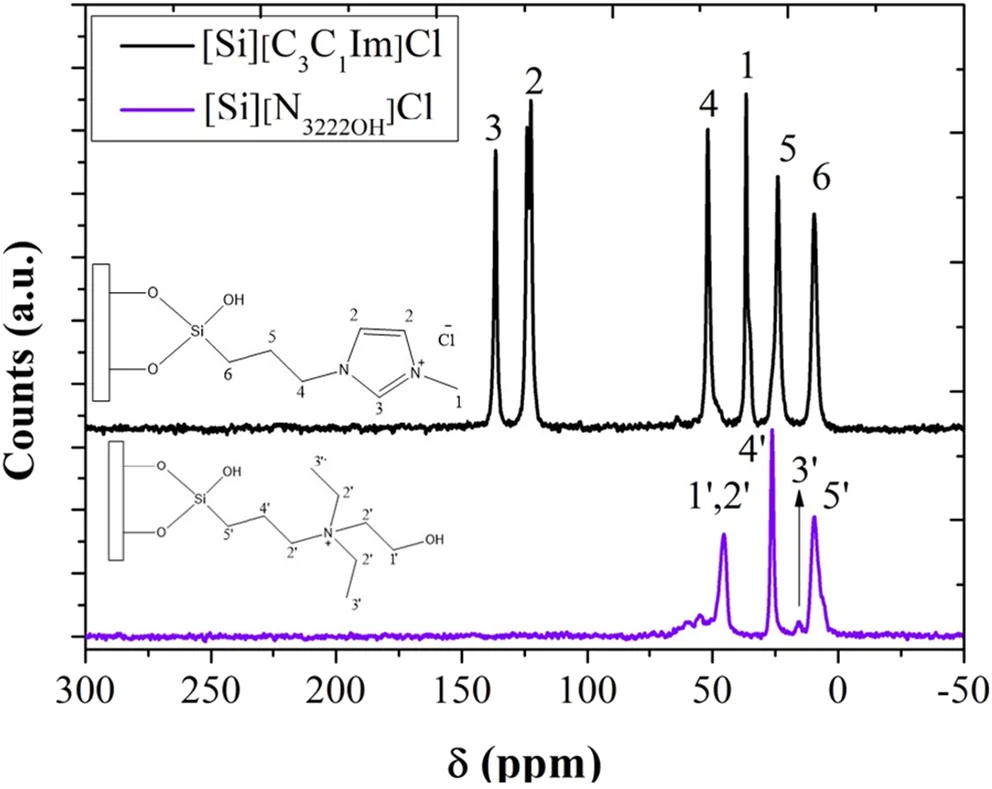
^13^C NMR solid-state spectrum of [Si]­[N_3222OH_]Cl (purple line) and [Si]­[C_3_C_1_Im]Cl (black line).

The results of the elemental analysis of each SIL are provided in Table 1. The carbon percentages are 6.11% and 8.36% for the SILs [Si]­[N_3222OH_]Cl and [Si]­[C_3_C_1_Im]­Cl, respectively. The nitrogen content in [Si]­[N_3222OH_]Cl and [Si]­[C_3_C_1_Im]Cl is 0.44% and 2.83%, respectively, indicating thus the silica functionalization with ILs.

**Table 1:** C, H, and N Weigh Fraction Percentage, Specific Surface Area (*S*_BET_), and IL Bonding Density of SILs

	% Carbon	% Hydrogen	% Nitrogen	*S*_BET_ (m^2^ g^–1^)	Bonding density (μmol m^–2^)
Silica	-	-	-	440.00	-
[Si][N_3222OH_]Cl	6.11 ± 0.01	1.41 ± 0.01	0.44 ± 0.00	343.16	0.71
[Si][C_3_C_1_Im]Cl	8.36 ± 0.01	1.68 ± 0.00	2.83 ± 0.00	212.25	2.30

The *S*_BET_ decreased from 440 m^2^ g^–1^ for pristine silica gel to 343.16 m^2^ g^–1^ and 211.25 m^2^ g^–1^ for SILs [Si]­[N_3222OH_]Cl and [Si]­[C_3_C_1_Im]­Cl, respectively. This reduction is mainly attributed to the partial occupation and/or blockage of silica pores by the grafted ionic liquid moieties, which decreases the accessible surface area and pore volume. Such behavior is commonly observed after surface functionalization of porous materials.^10,14^ The bonding density of SILs [Si]­[C_3_C_1_Im]Cl and [Si]­[N_3222OH_]Cl are 2.30 and 0.71 μmol m^–2^, respectively. For [Si]­[C_3_C1Im]­Cl, the bonding density differs from the value reported in the literature (2.33 μmol m^–2^),^21^ which can be attributed to differences in the synthesis conditions. In particular, the literature procedure employed a higher amount of precursor (ca. 0.061 mol, corresponding to 5 mL of *N*-methylimidazole), whereas in the present work 0.035 mol was used for the same amount of [Si]­[C_3_]­Cl.

### 3.2 Adsorption Kinetics and Isotherms

Adsorption kinetics, diffusion mechanisms, and equilibrium isotherms of citric acid on the SILs were investigated to provide insights for the potential application of these materials in fixed-bed column processes for the purification of citric acid produced industrially via glycerol bioconversion. As shown in Figure 2, the interaction kinetics between citric acid and the SILs [Si]­[N_3222OH_]Cl and [Si]­[C_3_C_1_Im]Cl reveal a rapid adsorption stage, with equilibrium being approached within approximately 1 min of contact time. After this initial uptake, adsorption increases slightly up to about 30 min. Subsequently, both SILs exhibit partial desorption, resulting in a gradual decrease in adsorption that stabilizes at lower values up to 180 min.

**Figure 2:**
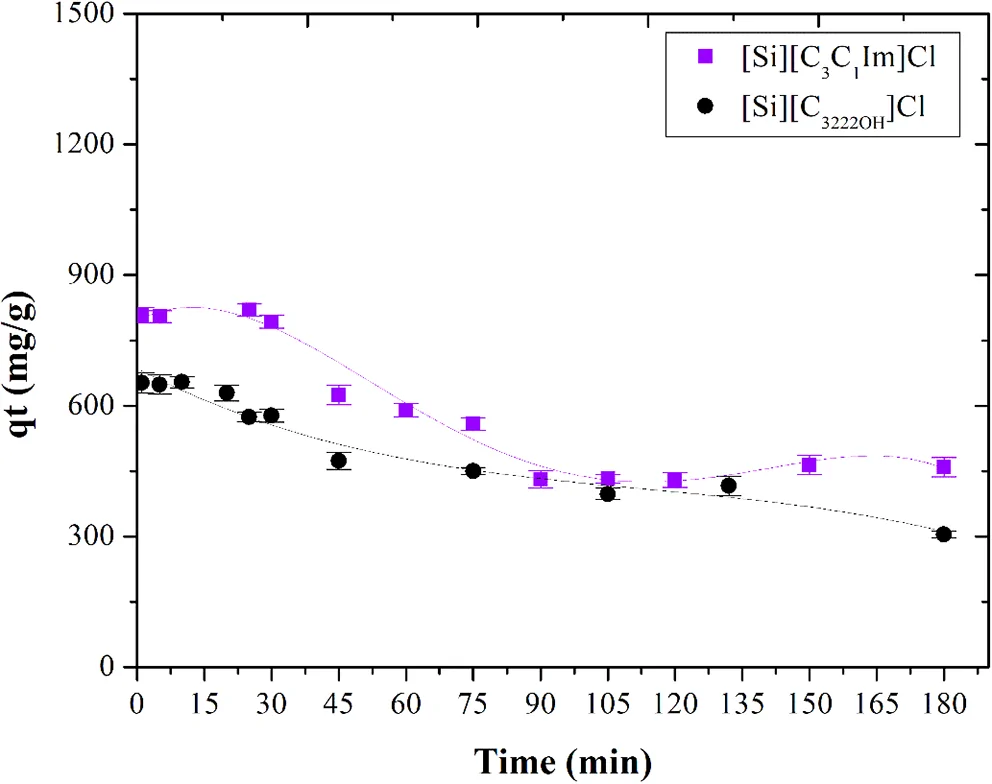
Adsorption kinetic curves for citric acid with [Si]­[N_3222OH_]Cl and [Si]­[C_3_C_1_Im]Cl at 25 °C. Experimental conditions: 100 mL of a citric acid solution (10 g·L^–1^) for 500 mg of SILs ([Si]­[N_3222OH_]Cl or [Si]­[C_3_C_1_Im]­Cl) at 120 rpm.

From a process perspective, the rapid adsorption response is advantageous for prospective fixed-bed column applications, particularly under high-flow conditions, which is desirable for industrial-scale purification processes. Nevertheless, the results also indicate limited stability in the interaction between citric acid and the SILs over time. This behavior is likely associated with the molecular structure of the materials. Although both SILs display high adsorption capacities for citric acid, they are reported to exhibit relatively high points of zero charge (PZC), characteristic of positively charged surfaces, which may influence the stability of the adsorption interactions.^10,11^

Citric acid is a weak organic acid that does not fully ionize in aqueous media. This behavior may lead to competitive interactions between protonated water molecules and the positively charged silica surface, thereby reducing the stability of the electrostatic interactions responsible for adsorption. Despite this effect, the SILs exhibited high adsorption capacities, with *q*_e_ (experimental equilibrium concentration of the sorbate in the solid phase), values reaching up to 770 mg·g^–1^ for [Si]­[N_3222OH_]Cl and 804 mg·g^–1^ for [Si]­[C_3_C_1_Im]­Cl.

Based on the experimental kinetic data, the adsorption behavior was analyzed using the pseudo-first-order, pseudo-second-order, intraparticle diffusion, and Boyd kinetic models, as summarized in Table 2. Among the models evaluated, the pseudo-second-order model provided the best fit to the experimental data, presenting the highest correlation coefficients for both SILs, with values of 0.9651 and 0.9855 for [Si]­[N_3222OH_]Cl and [Si]­[C_3_C_1_Im]­Cl, respectively. According to this model, the adsorption of citric acid onto [Si]­[N_3222OH_]­Cl and [Si]­[C_3_C_1_Im]Cl is likely governed by interactions involving two active sites on the adsorbent surface.^22^ This behavior is consistent with previous studies involving similar SILs, for which the pseudo-second-order model has also been reported to provide the best fit for the adsorption of other compounds, including acetylsalicylic acid,^10^ pesticides,^11^ and sodium diclofenac.^9^

**Table 2:** Adsorption Parameters Obtained from the Fitting of Adsorption Kinetic Experimental Data with the Pseudo First-Order, Pseudo Second-Order Models, Intra-Particle Diffusion and Boyd, and the Respective Correlation Coefficients for Citric Acid with [Si]­[N_3222OH_]Cl and, [Si]­[C_3_C_1_Im]­Cl

	[Si][N_3222OH_]Cl	[Si][C_3_C_1_Im]Cl
Pseudo First-Order		
*q*_e_ (mg·g^–1^)	653.08	853.27
*K*_1_ (min^–1^)	–0.0045	–0.0055
*R* ^2^	0.9638	0.8642
Pseudo Second-Order		
*K*_2_ (g·mg^–1^ min^–1^)	0.0030	0.0023
*q*_e_ (mg·g^–1^)	–0.0296	–0.0188
*R* ^2^	0.9651	0.9855
Intraparticle Diffusion		
*K*_D_ (mg·g^–1^ min^–1/2^)	–29.7645	–38.8417
*C* (mg·g^–1^)	719.94	902.89
*R* ^2^	0.9414	0.8247
Boyd		
*D*	–1.2519	–1.0884
*B* (min^–1^)	0.0042	0.0052
*R* ^2^	0.9391	0.8442

With regard to comparisons with other adsorption systems reported in the literature, the SILs investigated in this study exhibit kinetic behavior similar to that observed for related materials. For instance, silica supported with 1-methyl-3-propylimidazolium chloride used for the adsorption of acetylsalicylic acid reached equilibrium within approximately 20 min and remained stable for up to 180 min.^10^ Similarly, the same SIL achieved equilibrium after about 15 min during the adsorption of sodium diclofenac and maintained stable adsorption up to 180 min.^9^

The experimental adsorption isotherm data obtained at 25 °C were fitted using the nonlinear Langmuir, Freundlich, and Dubinin–Radushkevich models, as shown in Figure 3. Based on the experimental results, no saturation of the SILs was observed up to a citric acid concentration of 25 g·L^–1^, with experimental *q*_max_ values of 193 mg·g^–1^ for [Si]­[N_3222OH_]Cl and 208 mg·g^–1^ for [Si]­[C_3_C_1_Im]­Cl. Higher adsorption capacities were observed in the kinetic experiments, where [Si]­[N_3222OH_]Cl presented *q*_e_ values ranging from 305 to 654 mg·g^–1^, while [Si]­[C_3_C_1_Im]Cl showed *q*_e_ values between 419 and 829 mg·g^–1^. This difference is attributed to the larger adsorbent mass used in the kinetic experiments (500 mg of SIL), which required a higher solvent volume to allow aliquot withdrawal without causing a statistically significant reduction in the initial volume (<15%). The increased adsorbent mass results in a higher number of available functional groups, which likely enhances adsorbent–adsorbate interactions and contributes to the higher adsorption capacities observed.

**Figure 3:**
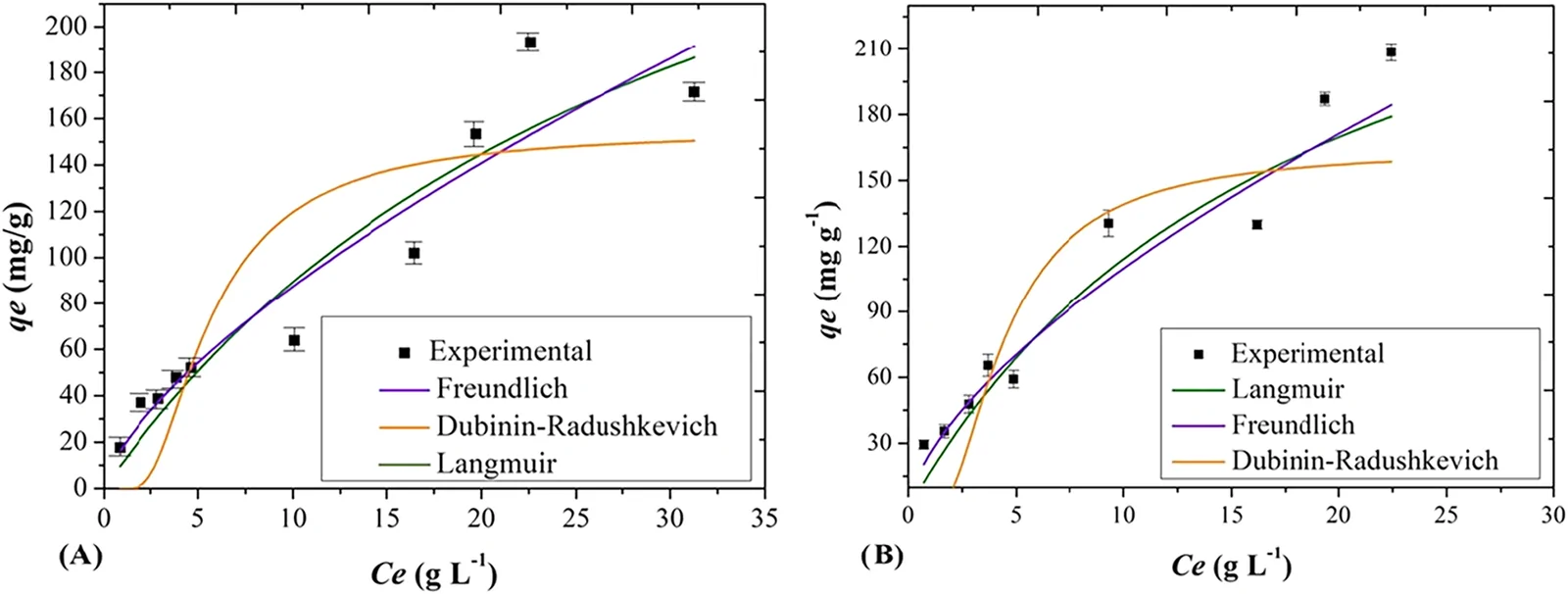
Adsorption isotherms for citric acid with [Si]­[N_3222OH_]Cl and [Si]­[C_3_C_1_Im]Cl at 25 °C. Experimental conditions: 5 mL of solutions with concentrations of citric acid ranging from 0.1 to 30 g·L^–1^, 50 mg of SILs ([Si]­[N_3222OH_]Cl or [Si]­[C_3_C_1_Im]­Cl), for 30 min. (A) [Si]­[N_3222OH_]Cl and (B) [Si]­[C_3_C_1_Im]­Cl.

To compare the adsorption performance of citric acid with other materials reported in the literature,^23–27^ the experimental *q*_e_ values obtained at 30 min from the kinetic experiments were considered (Table 3). As shown, the SILs evaluated in this study exhibit adsorption capacities among the highest reported. Furthermore, the performance of these SILs may be further enhanced under column adsorption conditions, where a substantially larger adsorbent mass is typically employed, thereby favoring stronger adsorbent–adsorbate interactions.

**Table 3:** Adsorption Capacities of Citric Acid with Different Adsorbents

Adsorbent	Capacity (mg·g^–1^)	References
Activated carbon	10.86	^23^
*Hevea brasiliensis* sawdust (activated carbon)	84.53	^24^
Microwave-assisted char synthesized from raw banana peel	147.06	^25^
*P. lentiscus* leaves (activated with NaOH)	666.67	^26^
Poly(4-vinylpyridine)	1140	^27^
[Si][N_3222OH_]Cl	577	This work
[Si][C_3_C_1_Im]Cl	793	This work

Among the models evaluated, the Freundlich model provided the best fit for the adsorption data of both SILs, as shown in Table 4. These findings are consistent with previous studies, where similar SILs also exhibited better agreement with the Freundlich model for the adsorption of different compounds.^9–11,21^ The predominance of this model suggests that the adsorption process occurs on heterogeneous surfaces and involves multilayer adsorption.^28^ This observation is also consistent with the results obtained from the pseudo-second-order kinetic model, which indicates that the adsorption process likely involves multiple active sites. Such behavior may facilitate multilayer adsorption and possibly overlapping interactions between adsorbate molecules on the SIL surface.

**Table 4:** Adsorption Parameters Obtained from the Fit of Adsorption Isotherms Experimental Data Isotherms with the Freundlich, Langmuir, and Dubinin–Radushkevich Models, and the Respective Correlations Coefficients for Citric Acid with [Si]­[N_3222OH_]Cl and [Si]­[C_3_C_1_Im]­Cl

	[Si][N_3222OH_]Cl	[Si][C_3_C_1_Im]Cl
Langmuir		
*q*_max_ (mg·g^–1^)	388.73	361.26
*K*_L_ (L·g^–1^)	0.0338	0.0515
*R* ^2^	0.8951	0.9306
*R*_L_	0.5967	0.4926
Freundlich		
*K*_F_ (L·g^–1^)	17.4698	25.3826
*n*	1.3720	1.5122
1/*n*	0.7289	0.6613
*R* ^2^	0.9295	0.9454
Dubinin–Radushkevich		
*q*_max_ (mol g^–1^)	258.79	179.27
β (mol^2^·kJ^–2^)	196.25	20.89
*E*	0.0505	0.1547
*R* ^2^	0.4422	0.7935

According to the constants obtained by the Freundlich model, the values obtained for *n* for the SILs [Si]­[N_3222OH_]Cl and [Si]­[C_3_C_1_Im]Cl were 1.3720 and 1.5122, respectively. According to the model’s postulates, if the value of *n* is between 1 and 10, the interaction between the adsorbent and the adsorbate is greater than that between the adsorbent and the solvent, which indicates that adsorption is favorable

Regarding the parameters obtained from the Freundlich model, the *n* values determined for the SILs [Si]­[N_3222OH_]Cl and [Si]­[C_3_C_1_Im]Cl are 1.3720 and 1.5122, respectively. Based on the assumptions of the Freundlich model, *n* values between 1 and 10 indicate favorable adsorption,^29^ suggesting that the interactions between the adsorbent and the adsorbate are stronger than those between the adsorbent and the solvent.

The Freundlich constant *K*_F_ was further used to estimate the thermodynamic parameters of the adsorption process, as presented in Table 5. The negative Gibbs free energy changes indicate that the adsorption process is spontaneous^25^ for both SILs, with values of −7.09 and −8.01 kJ·mol^–1^ for [Si]­[N_3222OH_]Cl and [Si]­[C_3_C_1_Im]­Cl, respectively. The enthalpy changes, −8.05 and −9.10 kJ·mol^–1^ for [Si]­[N_3222OH_]Cl and [Si]­[C_3_C_1_Im]­Cl, respectively, suggest that the adsorption mechanism is predominantly governed by physisorption, since adsorption enthalpies up to approximately 25 kJ·mol^–1^ are typically associated with physical adsorption processes.^30^ The negative entropy changes (−3.25 and −3.67 J·mol^–1^·K^–1^ for [Si]­[N_3222OH_]Cl and [Si]­[C_3_C_1_Im]­Cl, respectively) indicate a decrease in randomness at the solid-solution interface during adsorption.^29^ This behavior suggests that no significant structural changes occur in the adsorbent during the process, while the system becomes more ordered upon citric acid adsorption.

**Table 5:** Thermodynamic Parameters for Citric Acid Adsorption Process with [Si]­[N_3222OH_]Cl and [Si]­[C_3_C_1_Im]­Cl^a^

	[Si][N_3222OH_]Cl	[Si][C_3_C_1_Im]Cl
Δ*G*° (kJ·mol^–1^·K ^–1^)	–7.09	–8.01
Δ*H*° (kJ·mol^–1^)	–8.05	–9.10
Δ*S*° (J·mol^–1^·K ^–1^)	–3.25	–3.67

a Calculations performed based on the equation Δ*G*° = −*RT* Ln KF,^31^ where *R* is the universal gas constant, equal to 8.314 J·mol^–1^·K^–1^, KF (L·mol^–1^) is the Freundlich constant, and *T* is the temperature of 298 K.

Negative values of Gibbs free energy are commonly observed in adsorption processes involving different adsorbent materials. Similar behavior has been reported in the literature; for example, a study on the adsorption of citric acid using microwave-assisted activated carbon synthesized from raw banana peel reported a negative Gibbs free energy change of −3.99 kJ·mol^–1^ at 30 °C,^25^ confirming the spontaneous nature of the adsorption process.

Regarding the effect of temperature (Figure 4), no significant variation is observed in the adsorption of citric acid over the temperature range studied for either SIL. For [Si]­[N_3222OH_]­Cl, adsorption efficiencies range from 82 to 89%, while for [Si]­[C_3_C_1_Im]­Cl values between 55 and 57% are obtained. This behavior may be explained by the relatively low enthalpy changes discussed above. Although the adsorption process is exothermic, the magnitude of the enthalpy change is small, suggesting that the amount of energy released is insufficient to significantly influence adsorption within the investigated temperature range. From an application perspective, this result is advantageous for industrial processes, as it indicates that small temperature fluctuations within the typical operational range of a fermentation process would have minimal impact on adsorption performance. Consequently, no additional temperature adjustment of the fermentation broth would be required for the purification of citric acid.

**Figure 4:**
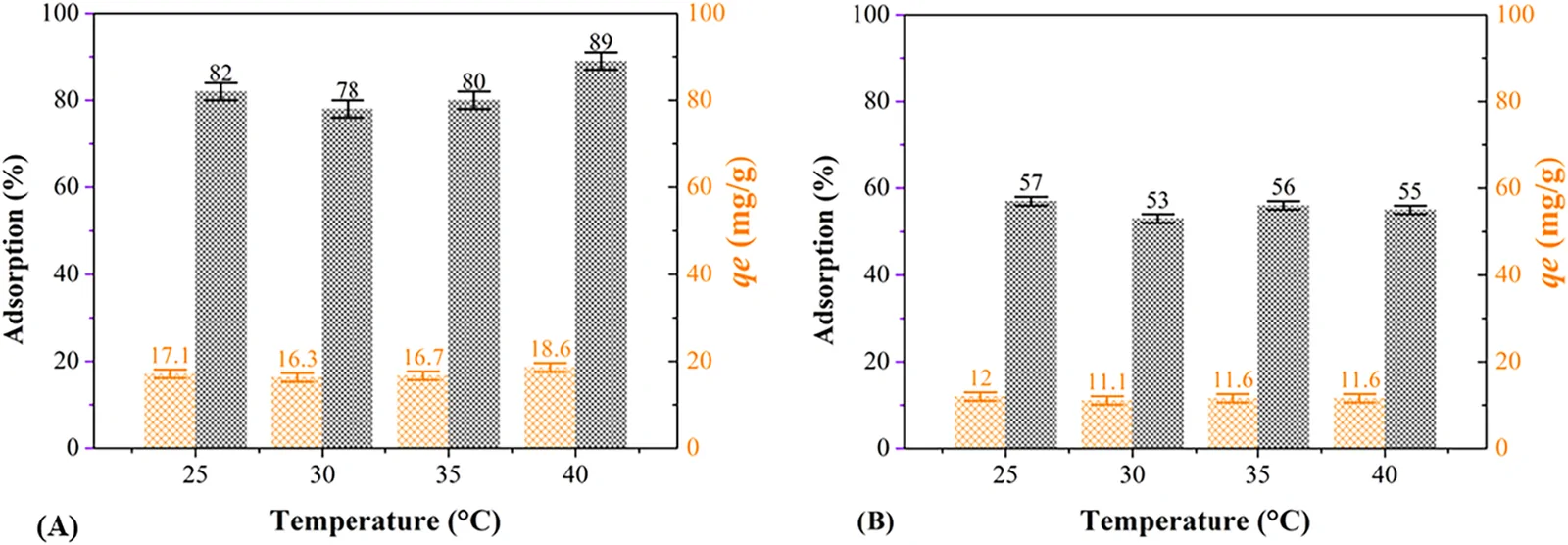
Temperature variation adsorption for citric acid with [Si]­[N_3222OH_]Cl and [Si]­[C_3_C_1_Im]Cl at different temperatures. Experimental conditions: 50 mg SIL; 1 mL of citric acid solution (10 g·L^–1^), 30 min, at temperatures 25, 30, 35, and 40 °C. (A) [Si]­[N_3222OH_]Cl and (B) [Si]­[C_3_C_1_Im]­Cl.

In contrast, other adsorbent systems reported in the literature exhibit a different behavior. For example, in the adsorption of citric acid using microwave-assisted activated carbon derived from raw banana peel, a much higher enthalpy change (95.818 kJ·mol^–1^) was reported, indicating an endothermic process.^25^ In that case, adsorption increased with temperature in the range of 30–50 °C. These observations support the hypothesis that the magnitude of the enthalpy change is directly related to the sensitivity of the adsorption process to temperature variations.

Citric acid fermentation typically occurs at a pH close to 2, where the acid molecules predominantly remain in their protonated form. In conventional recovery processes, calcium hydroxide is added to convert the protonated acid into an insoluble calcium citrate salt, enabling its separation from the fermentation broth.^27^ Consequently, adsorption-based purification strategies may be applied either directly after fermentation under acidic conditions or after the primary recovery step at moderately higher pH values.

As shown in Figure 5, [Si]­[N_3222OH_]Cl exhibits its best performance at pH 3, reaching an adsorption efficiency of 97%. This result suggests that the adsorbent interacts more effectively with the protonated form of citric acid, likely through hydrogen-bonding interactions. This behavior is consistent with the acid dissociation constants of citric acid (p*K*_a1_ = 3.13, p*K*_a2_ = 4.76, and p*K*_a3_ = 6.40),^32^ indicating that at pH 3 the molecule is predominantly present in the fully protonated form, (HOOCCH_2_)_2_C­(OH)­COOH.

**Figure 5:**
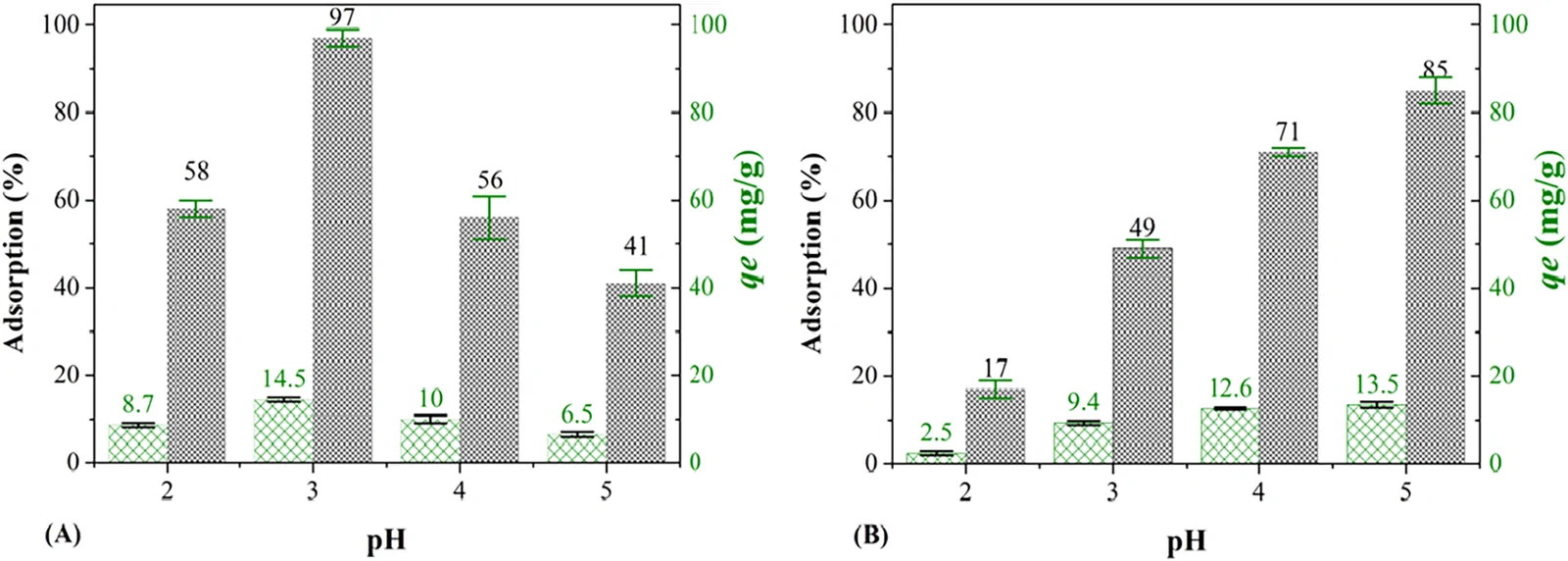
Adsorption of citric acid onto SILs with pH variation. Experimental conditions: 50 mg SIL; 1 mL of citric acid solution (10 g·L^–1^), for 30 min at 25 °C, at pH values of 2, 3, 4, and 5. (A) [Si]­[N_3222OH_]Cl and (B) [Si]­[C_3_C_1_Im]­Cl.

In contrast, [Si]­[C_3_C_1_Im]­Cl shows its highest adsorption performance at pH 5, achieving approximately 85% adsorption. This behavior suggests a different adsorption mechanism, possibly associated with electrostatic interactions involving the partially deprotonated citrate species formed at this pH. The presence of multiple anionic forms arising from the stepwise deprotonation of the triprotic citric acid molecule may allow interaction with multiple exchange sites on the adsorbent surface.

In the work reported by Van den Berg et al.,^27^ different adsorption mechanisms for citric acid were proposed depending on its degree of deprotonation. When citric acid is deprotonated, adsorption predominantly occurs through strong ionic exchange interactions. In contrast, when the molecule remains in its protonated form, adsorption is mainly governed by hydrogen-bonding interactions. Their findings are consistent with the observations of the present work. Specifically, the Amberlite IRA-900 resin showed higher adsorption at pH 4 compared with pH 2 and pH 7, indicating that intermediate pH conditions favor the interaction between citric acid species and the adsorbent surface.

### 3.3 Adsorption Efficiency in Consecutive Adsorption and Desorption Cycles

As shown in Figure 6A, [Si]­[N_3222OH_]Cl exhibits a progressive decrease in citric acid adsorption efficiency over consecutive adsorption cycles, with values of 74.3%, 54.6%, 40.1%, and 36.9%. This trend suggests a gradual loss of adsorption toward citric acid during repeated use. Nevertheless, the material demonstrated complete desorption (100%) of citric acid in all cycles, which is advantageous for citric acid recovery and purification processes. In contrast, [Si]­[C_3_C_1_Im]Cl (Figure 6B) shows an initial increase in adsorption efficiency from the first to the second cycle, rising from 48.9% to 88.5%, followed by a decrease in the subsequent cycles to 62.8% and 53.7%. Regarding desorption, the efficiency increased from 43.8% in the first cycle to 74.5% in the second cycle and then remained relatively stable in the following cycles, with values of 75.7% and 78.9%. These results suggest that the interactions between citric acid and [Si]­[N_3222OH_]Cl are weaker than that with [Si]­[C_3_C_1_Im]­Cl. The presence of the *N*-methylimidazolium group in [Si]­[C_3_C_1_Im]Cl may promote stronger or more selective interactions with citric acid, potentially enhancing adsorption performance. Despite the observed loss of adsorption performance and limited robustness upon reuse, the developed SILs exhibit high desorption efficiencies and operational simplicity, which, together with their low cost, highlight their potential as practical materials for citric acid recovery, particularly in single-use or short-cycle separation processes.

**Figure 6:**
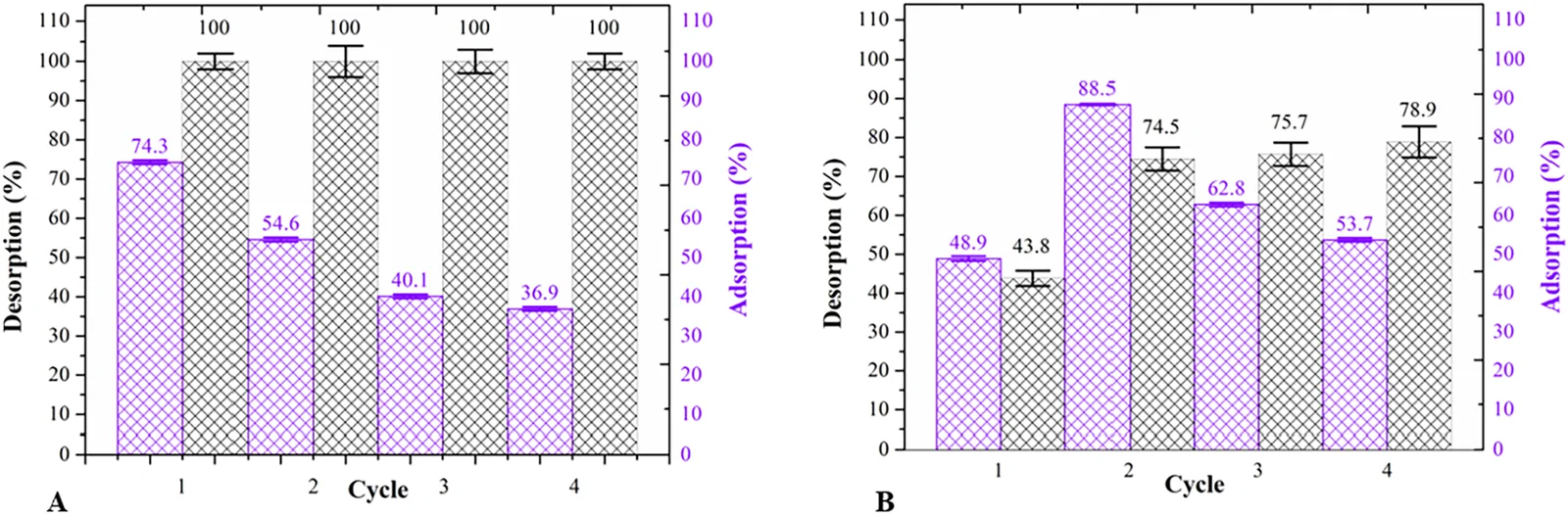
Adsorption efficiency of citric acid onto SILs, consecutive adsorption and desorption cycles corresponding to the material recycling. (A) [Si]­[N_3222OH_]Cl and (B) [Si]­[C_3_C_1_Im]­Cl.

### 3.4 Recovery of Citric Acid from the Fermentation Broth

In the citric acid adsorption experiments using the fermented broth (Figure 7), [Si]­[C_3_C_1_Im]Cl exhibited slightly higher adsorption and desorption efficiencies, reaching 11.2% and 85.7%, respectively, compared with [Si]­[N_3222OH_]­Cl, which showed 10.3% for the adsorption and 53.8% for the desorption. Although the adsorption values are relatively low compared to the previously discussed assays, it is important to note that these preliminary tests were conducted using cell-free raw fermentation broth without any prior purification or conditioning steps. The presence of residual metabolites, salts, and other organic compounds in the broth may have competed with citric acid for the available adsorption sites or partially blocked the pores of the SILs, thereby limiting adsorption performance. Despite this complexity, the high desorption efficiencies, particularly for [Si]­[C_3_C_1_Im]­Cl, highlight the promising selectivity of SILs toward citric acid, even in complex fermentation matrices. Moreover, the adsorption studies conducted in this work demonstrated rapid adsorption kinetics and the capacity of the SILs to accumulate citric acid on their surfaces, characteristics that are advantageous for continuous separation processes. These features indicate that the materials may be suitable for application in fixed-bed column systems operating at relatively high flow rates.

**Figure 7:**
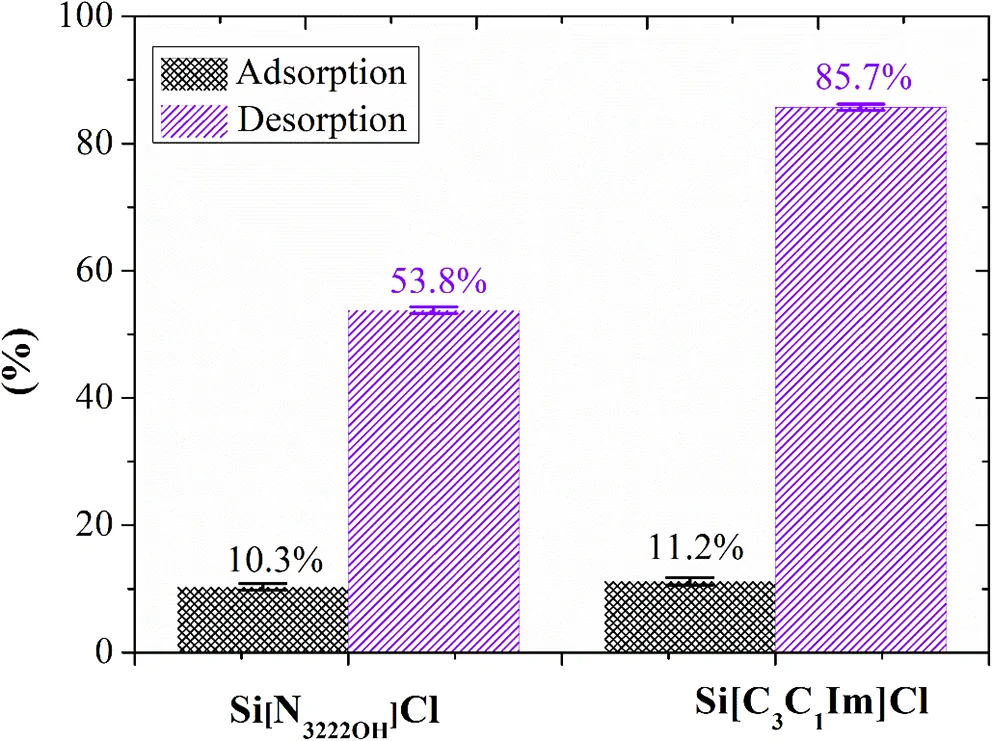
Adsorption and desorption of citric acid from the fermented broth onto SILs at 25 °C. Experimental conditions: 50 mg; 5 mL of fermented broth containing citric acid (9 g·L^–1^), for 30 min at 25 °C.

Overall, the obtained results provide a promising basis for future pilot-scale studies involving adsorption of citric acid directly from fermentation broths in fixed-bed columns. Further investigations should focus on optimizing the process conditions, evaluating the influence of competing salts present in the broth, and integrating SIL-based adsorption with primary citric acid recovery steps to enhance overall purification efficiency.

## 4. Conclusions

The SILs [Si]­[N_3222OH_]Cl and [Si]­[C_3_C_1_Im]Cl were successfully synthesized, characterized, and evaluated as adsorbent materials for the recovery of citric acid, aiming to develop an alternative strategy for its industrial recovery. Both materials demonstrated rapid adsorption kinetics, and high adsorption capacities were achieved, reaching 577 and 793 mg·g^–1^ for [Si]­[N_3222OH_]Cl and [Si]­[C_3_C_1_Im]­Cl. These results place the studied SILs among the most effective adsorbents reported for citric acid recovery.

The adsorption behavior was well described by the pseudo-second-order kinetic model, while the equilibrium data were best fitted by the Freundlich isotherm, indicating adsorption on heterogeneous surfaces and the occurrence of multilayer interactions. Thermodynamic analysis further revealed that the adsorption process is spontaneous, as indicated by the negative Gibbs free energy values. The relatively low enthalpy changes suggest that the adsorption mechanism is predominantly governed by physisorption, while the negative entropy values indicate a decrease in randomness at the solid–solution interface without significant structural changes in the adsorbent.

Reusability tests demonstrated that both SILs maintain the desorption performance over multiple cycles, highlighting the potential for citric acid recovery and material regeneration. Preliminary experiments using real fermentation broth also confirmed the ability of the SILs to interact selectively with citric acid even in complex media, with particularly promising desorption efficiencies.

Overall, the obtained results represent a basis for the application of SIL-based materials in citric acid recovery processes. The combination of rapid adsorption kinetics, high adsorption capacity, and efficient regeneration suggests that these materials are promising candidates for continuous separation systems, such as fixed-bed columns operating at relatively high flow rates. Future studies should focus on optimizing adsorption conditions, evaluating the influence of competing species present in fermentation broths, integrating the adsorption step with primary recovery processes, and assessing performance in pilot-scale continuous systems.
